# MCP-1, CCR2 and CCR5 polymorphisms in Tunisian patients with atopic asthma

**DOI:** 10.1186/1479-5876-9-S2-P10

**Published:** 2011-11-23

**Authors:** Tarak Dhaouadi, Imen Sfar, Hajer Aounallah-Skhiri, Saloua Jendoubi-Ayed, Mohamed Amri, Hend Bouacha, Taieb Ben Abdallah, Khaled Ayed, Yousr Gorgi

**Affiliations:** 1 Laboratory of Research in Renal Transplantation and Immunopathology (LR03SP01), Charles Nicolle Hospital, Tunis El Manar University, Tunis, Tunisia; 2 National Institute of Public Health, Tunis, Tunisia; 3 Pneumonology Dept, Charles Nicolle Hospital, Tunis, Tunisia

## Background

Chemokines and their receptors play an important in the late inflammatory stage occurring in asthma.

## Objective

We aimed to investigate polymorphisms of MCP-1 (CCL2), CCR2 and CCR5 which can modify qualitatively and/or quantitatively their production and thus influence both susceptibility to asthma and its clinical and biological features.

## Patients and methods

MCP-1 (A/G -2518), CCR2 (+/64I), CCR5 (G/A -59029) and CCR5 (Δ32) polymorphisms were detected by PCR in 107 Tunisian patients with asthma and 169 healthy controls.

## Results

We found no significant association between any of the four investigated polymorphisms and asthma. Nevertheless the haplotype MCP1∗AG**/**CCR2∗+/+ was significantly less frequent in patients (20,5%) than in controls (32,5%) p=0,03 OR=0,54 95% CI: 0,29-0,98. While no difference observed in CCR2/CCR5 haplotypes between patients and controls.

Analysis of polymorpohisms with clinical and biological features showed a non significant decrease of the frequency of MCP-1∗G and CCR2∗64I alleles in patients with severe disease, moreover the concomitant presence of MCP-1∗G/CCR2∗64I alleles was less frequent in severe forms (4,34%) than in other patients (12%) but the difference was no longer significant p=0,27. No associations were observed between the four polymorphisms and the presence of atopic rhinitis or atopic conjunctivitis and an elevated rate of serum IgE over 200 UI/ml.

## Conclusion

Polymorphisms of MCP-1 and its receptor CCR2 seem to be involved in disease-susceptibility to asthma in Tunisian; nevertheless they could be protective against its severe forms.

